# Effects of fibular strut augmentation for the open reduction and internal fixation of proximal humeral fractures: a systematic review and meta-analysis

**DOI:** 10.1186/s13018-022-03211-4

**Published:** 2022-06-21

**Authors:** Wei Nie, Zhaojun Wang, Fei Gu, Shizhuang Xu, Yang Yue, Anze Shao, Kefu Sun

**Affiliations:** Department of Orthopedic Surgery, Lianyungang 2nd People’s Hospital, No. 41 Hailian East Road, Haizhou District, Lianyungang, 222000 Jiangsu China

**Keywords:** Fibular strut augmentation, Proximal humeral fracture, Open reduction and internal fixation, Locking plate, Review, Meta-analysis

## Abstract

**Background:**

There is still a lack of remarkable consensus regarding the clinical efficacy of the application of fibular strut augmentation (FSA) combined with a locking plate for proximal humeral fractures. A systematic review and meta-analysis to assess the effect of the use of FSAs in open reduction and internal fixation of proximal humeral fractures was conducted.

**Methods:**

A literature search was conducted in PubMed, Embase, Cochrane, Web of Science Core Collection, and ClinicalTrials.gov to identify trials that compared the clinical outcomes of proximal humeral fractures treated using a locking plate with or without FSA. The primary outcome measures were postoperative complications, radiographical findings, functional recovery scores, and postoperative range of motion (ROM). Data were pooled and analysed using a random-effects model based on the Der Simonian and Laird method.

**Results:**

Eight studies involving 596 participants were included for further analysis. Compared with using a locking plate independently, the additional application of FSA was associated with the likelihood of lower risk of overall complications (OR 0.37; 95% CI 0.22–0.65; *I*^*2*^ = 12.22%; 95% PI 0.14–0.98) and the rate of patients with orthopaedic complications (OR 0.48; 95% CI 0.25–0.92; *I*^*2*^ = 7.52%; 95% PI 0.16–1.45), less changes in postoperative humeral head height (MD − 2.40; 95% CI − 2.49 to − 2.31; *I*^*2*^ = 0.00%; 95% PI − 2.61 to − 2.20) and the neck–shaft angle (MD − 6.30; 95% CI − 7.23 to − 5.36; *I*^*2*^ = 79.32%; 95% PI − 10.06 to − 2.53), superior functional outcomes (Constant–Murley score: MD 5.07; 95% CI 3.40 to 6.74; *I*^*2*^ = 0.00%; 95% PI 2.361–7.78; American Shoulder and Elbow Surgeons Score: MD 5.08; 95% CI 3.67 to 6.49; *I*^*2*^ = 0.00%; 95% PI 1.98–8.18), and better postoperative ROM in terms of forward elevation and external rotation. However, the evidence regarding postoperative abduction was insufficient.

**Conclusion:**

Meta-analytic pooling of current evidence showed a significant association between the application of FSAs and favourable clinical outcomes in terms of postoperative complications, radiographical findings, functional recovery, and postoperative elevation and external rotation.

**Supplementary Information:**

The online version contains supplementary material available at 10.1186/s13018-022-03211-4.

## Background

The optimal treatment for proximal humeral fractures is an ongoing controversy, and the indications for each treatment option continue to evolve [1, 2]. Although nonoperative treatment is reported to be associated with acceptable outcomes, surgical intervention is still recommended especially for fractures with displacements greater than 1 cm or angular deformities great than 45° [3, 4]. Open reduction and internal fixation (ORIF) with a locking plate is considered to be a typical treatment option that could provide anatomical reduction and improved function [4]. However, early outcomes of ORIF demonstrated a distinct subset of complications and a high risk of hardware failure [5].

Newer data suggest that the lack of support for the medial column is the main contributor to the fixation failure, especially in geriatric patients with severe osteoporosis and persistent medial malalignment after reduction [6, 7]. In response to this, intramedullary fibular strut augmentation (FSA) was introduced as an adjuvant to the locking plate in an attempt to facilitate fracture reduction and enhance the mechanical stability of the medial column, thus decreasing postoperative complications and achieving optimal outcomes [8]. To date, only a limited number of comparative clinical studies have been performed and there is still a lack of consensus regarding its clinical efficacy [9, 10]. There is still no meta-analysis based on comparable studies to investigate the efficacy of FSAs in the surgical management of proximal humeral fractures. With the aim of generating evidence for or against the application of this technique, a meta-analysis was performed based on available literature that directly compared the outcomes of ORIF using the locking plate with and without FSA for proximal humeral fractures.

## Materials and methods

### Literature search

This systematic review was reported in accordance with the Preferred Reporting Items for Systematic Reviews and Meta-Analyses (PRISMA) statements [11] and was registered in the International Prospective Register of Systematic Reviews (PROSPERO #CRD42021230374). To identify related studies, a literature search was conducted in PubMed, Embase, Cochrane Library, Web of Science Core Collection, and ClinicalTrials.gov in September 2021, and an update was performed in March 2022. Additional articles were traced manually from reference lists of relevant studies. Articles published after 2008, when this technique was first described by Gardner et al. [8], and written in English were eligible. To improve the recall ratio, no special filter was used in the search strategy, and the Medical Subject Headings (MeSH) terms and Entry terms for the “Comparison” (e.g. locking plate, internal fixation, etc.) were not designed in the search strategy. The search strategy used in PubMed is tabulated in Additional file 1 and was tailored to other databases.

### Study selection and quality assessment

Studies that met the following criteria were assessed for further analyses: (1) evaluated adults aged at least 18 years with proximal humeral fractures; (2) directly compared the clinical results of proximal humeral fractures using a locking plate with or without FSA; and (3) reported outcomes of interest, including intraoperative blood loss, surgical duration, complications, radiographical findings, and functional recovery. Single group observational studies, and studies enrolling paediatric patients or cancer patients, reporting the same trial, having only abstracts or lacking data of interest were excluded. When the study did not provide the data in an appropriate form (e.g. “median, range” for continuous outcomes), the first choice was to contact the corresponding author to obtain the required data. If there was no response from the authors after two inquiries, the study would be excluded from further analyses.

The methodological quality of eligible studies was assessed and rated by two reviewers independently using the Newcastle–Ottawa Scale (NOS) for cohort studies [12], for cohort studies [12], which is based on a star scoring system with a maximum of nine stars to evaluate a study in three domains (including 8 items): selection of participants (a maximum of 4 stars), comparability of study groups (a maximum of 2 stars), and ascertainment of outcomes of interest (a maximum of 3 stars). Studies would be excluded if: (1) they were judged to be of poor quality as they received less than five stars according to the NOS; (2) there was a major defect such as a high rate of loss to follow-up (generally ≥ 20%) or a substantial lack of baseline comparability between groups, which would threaten the internal validity of the study. Discordant quality ratings were resolved by discussion or by consulting a third reviewer.

### Outcome measures and data collection

Recorded variables were as follows: authors, publication year, study design, sample size, patients demographics, trial duration, and clinical outcomes. To evaluate the effectiveness of FSA, the radiological findings, functional evaluation scores, postoperative shoulder range of motion (ROM) and major complications related to the surgeries were regarded as primary outcome measures. Other information such as intraoperative blood loss and surgical duration was also extracted and analysed.

### Statistical analysis

A random-effects model with the Der Simonian and Laird method was used, as the authors anticipated the instability of study variance due to differences in study characteristics. The binary outcomes were assessed by pooled odds ratios (ORs) and corresponding 95% confident intervals (CIs), and the unstandardized mean differences (MDs) and their 95% CIs were calculated for continuous outcomes. Interstudy heterogeneity was determined by the *I*^2^ indicator and was considered to be substantial with *I*^2^ more than 50%. The 95% prediction interval (PI) which could be regarded as a predictor of the treatment effect in a new study was also calculated as a reference [13]. Egger’s test was used to assess the risk of small-study effects, which may be suggestive, but not definitive of publication bias. All statistical analyses were calculated with Stata statistical software (version 16.0, StataCorp LLC), and statistical significance was defined as a *P* value less than 0.05 or a 95% confidence interval that could reject the null hypothesis.

## Results

### Characteristics and risk of bias of eligible studies

**Fig. 1:**
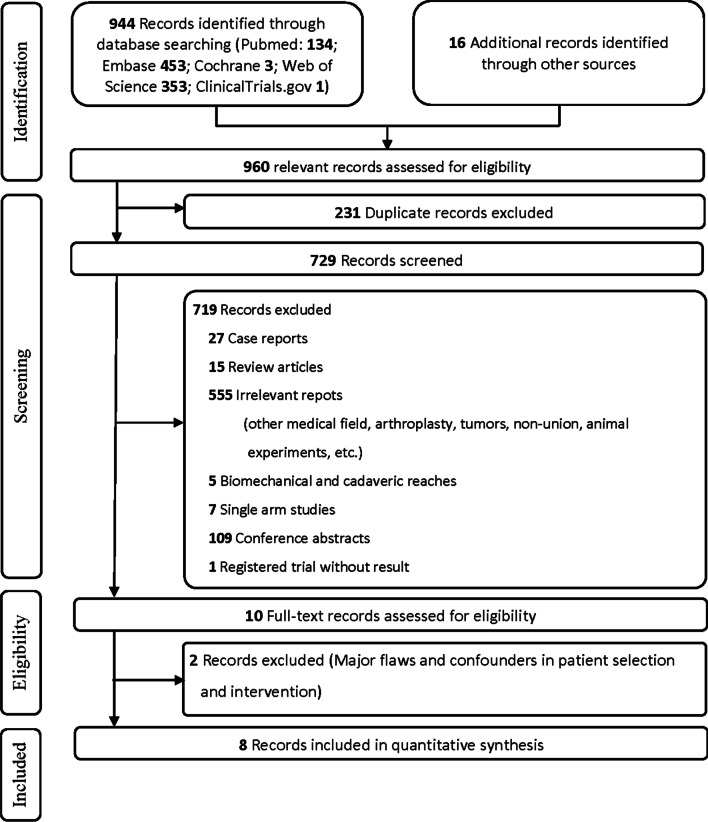
Flow diagram of the literature search and selection process

Two reviewers evaluated 944 articles from database searches and 16 citations from the reference list. After the initial screening of titles and abstracts, ten articles were considered for the next full-text assessment. No article was judged to be of poor quality. Two articles [14, 15] were excluded because other techniques that might be a potential confounding factor to the clinical result were used, thus leaving eight studies that were considered to be suitable for the inclusion in this review [9, 10, 16–21] (Fig. 1). Among them, one was a three-arm study including a group using another technique, and data from this group were not considered for statistical synthesis [20]. Finally, after excluding the cases lost to follow-up, data from 596 participants, with the average age ranging from 59.9 to 75.6 years and the average follow-up period ranging from 12 to 43 months, were collected and synthesized for the meta-analyses. All studies were retrospective and were performed in China (*n* = 5), South Korea (*n* = 2), and the USA (*n* = 1). The baseline information and characteristics of the studies are listed in Table 1.

**Table 1:** Characteristics and baseline of included studies

Study ID	County	Study type	Interventions	Sample size	Age (year)	Gender		Fracture type				Follow-up period	Result
								Neer classification system			AO classification system		
						Male	Female	2-part	3-part	4-part			
Chen 2018	China	retrospective	FSA + LCP	47	68.6	12	35		12	35		33.5	loss of reduction (>5 mm), functional outcomes (DASH, CMS) and complications
			LCP	42	69.12	15	27		10	32		35.2	
Cui 2019	China	retrospective	FSA + LCP	25	73.16	7	18		17	8		31.56	radiological changes in NSA and HHH, functional outcomes (DASH, CMS), ROM and complications
			LCP	35	72.46	11	24		25	10		32.23	
Davids 2020	America	retrospective	FSA + LCP	27	59.9 (19 to 90)	total 102: male 31, female 71		total 102: 2-part 53; 3-part 49				mean 17.6	functional outcomes (DASH, SST scores), VAS, ROM and complications
			LCP	75									
Kim 2020	South Korea;	retrospective	FSA + LCP	34	73.8 ± 5.80	13	21				A2 2; A3 13; B1 6; B2 11; B3 2	16.2 ± 3.74	average bone union time, mean NAS, CMS, ROM and complications
			LCP	29	73.8 ± 5.80	12	17				A2 3; A3 11; B1 4; B2 10; B3 1	18.6 ± 6.77	
Lee 2019	South Korea	retrospective	FSA + LCP	45	75.6 (55 to 87)	12	33	21	20	4		13.6 (12 to 17)	radiological changes in NSA and HHH, functional outcomes (DASH, CMS), VAS, ROM and complications
			LCP	52	73.3 (52 to 89)	14	38	25	22	5		14.2 (12 to 19)	
Wang 2019	China	retrospective	FSA + LCP	39	72.2 ± 5.9	16	23			39		19 (12 to 34)	the final CCD angle, functional outcome (ASES) and complications
			FSA + LCP	43	72.7 ± 5.6	14	29			43			
			LCP	46	72.5 ± 5.0	13	33			46			
Zhao 2019	China	retrospective	FSA + LCP	21	68.8 ± 6.3	11	10		14	7		12	radiological changes in NSA and HHH, functional outcomes (CMS, ASES, DASH), VAS and complications
			LCP	21	69.0 ± 7.2	12	9		15	6		12	
Tuerxun 2020	China	retrospective	FSA + LCP	41	64.1(34 to 89)	12	29	6	19	16		16.3	radiological changes in NSA and HHH, functional outcomes (CMS) and complications
			LCP	22	64.1(35 to 83)	9	13	3	10	9		16.3	

*ASES* American Shoulder and Elbow Surgeons Score, *CCD* Angle central column diaphyseal varus angle, *CMS* Constant–Murley score, *DASH* Disability of arm–shoulder–hand score, *FSA* fibular strut augmentation, *HHH* humeral head height, *LCP* locking plate, *NSA* neck–shaft angle, *SST* Simple Shoulder Test, *VAS* visual analogue scale

The results of NOS are summarized in Additional file 2. Although the cohorts were well-matched in each study, no trials were deemed high quality in all domains of NOS. The lack of adjustment to variables and inadequate follow-up periods were the most frequent deficiencies in trials.

### Postoperative complications

**Fig. 2:**
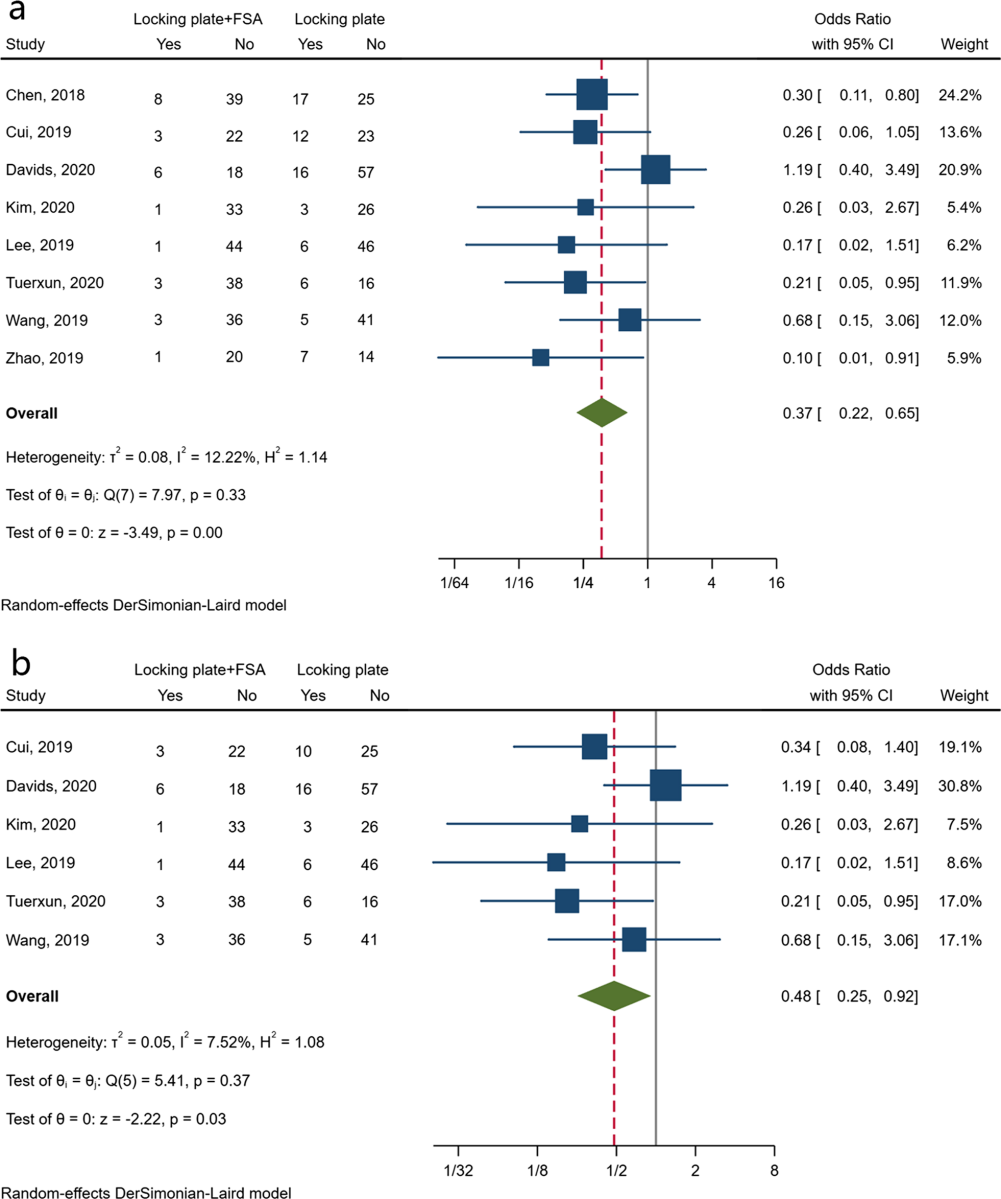
Forest plots of overall complications (**a**) and the rate of patients with orthopaedic complications (**b**)

Postoperative complications were reported in all eight studies. Compared with using a locking plate alone, the additional application of FSA was significantly associated with a lower risk of overall postoperative complications (OR 0.37; 95% CI 0.22–0.65; *I*^*2*^ = 12.22%; 95% PI 0.14–0.98) (Fig. 2a).

Concerning the variety of monitoring and reporting the kinds of complications between studies, we conducted a subsequent analysis on the rate of patients with orthopaedic complications related to surgical fixation, which mainly included varus malunion, screw perforation, failure of fixation, and humeral head avascular necrosis (HHAVN). Considering that one patient might have more than one complication and that there would be a strong link between these complications (e.g. a patient might have had varus malunion or humeral head collapse that led to a screw perforation), the complication rate was calculated as the number of patients with complications in each group, which was reported in six studies [10, 16–20]. Pooling the estimates confirmed a lower likelihood of the complication incidence associated with the application of FSA (OR 0.48; 95% CI 0.25–0.92; *I*^*2*^ = 7.52%; 95% PI 0.16–1.45) (Fig. 2b).

### Functional evaluations

**Fig. 3:**
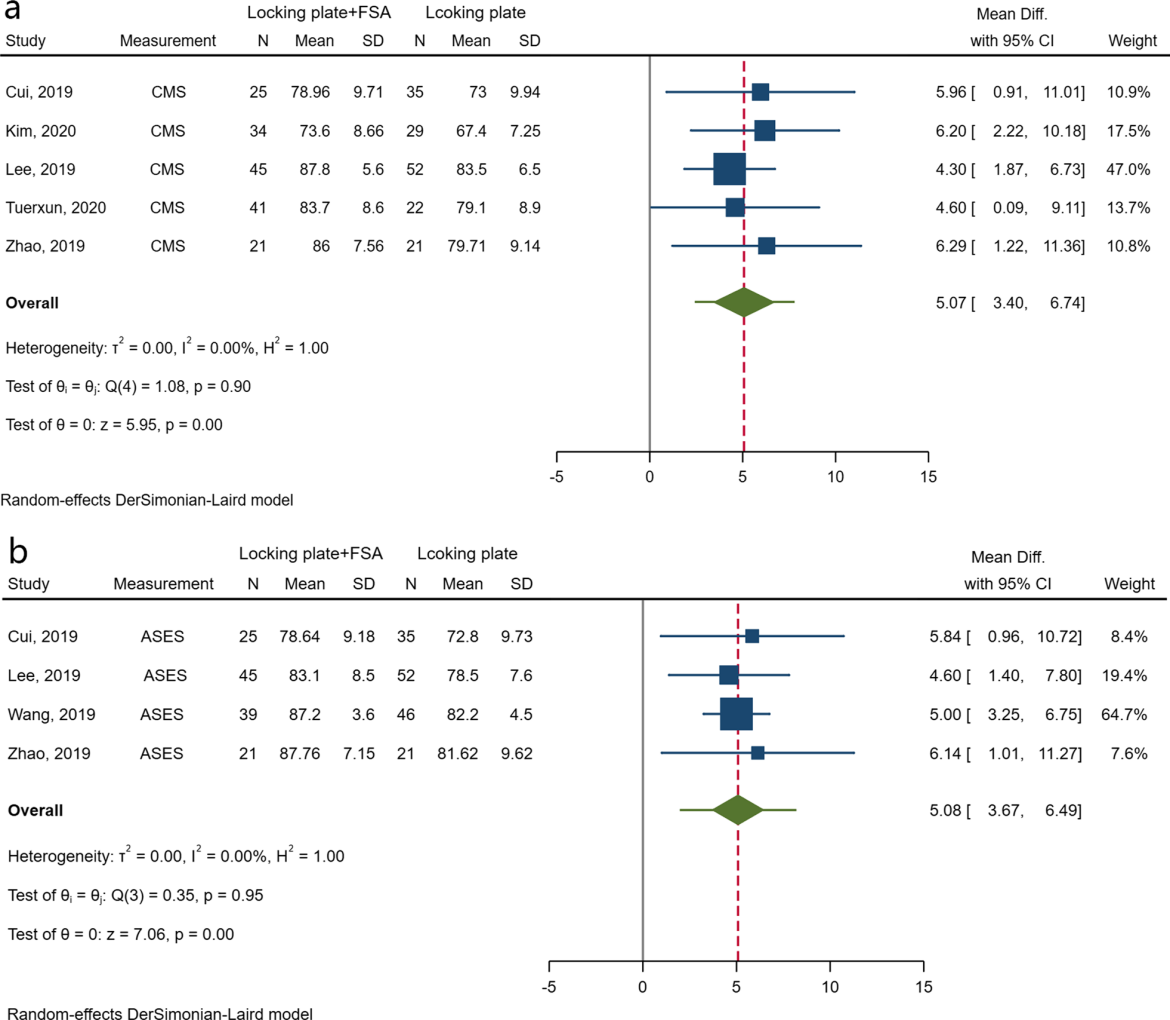
Forest plots of the results of CMS (**a**) and ASES (**b**)

Functional evaluation was performed in all eight trials. The measurement scales used in each trial varied, and most studies used more than one scale to assess the postoperative function, including the Constant score/Constant–Murley score (CMS) [22], American Shoulder and Elbow Surgeons (ASES) score [23], disability of arm–shoulder–hand (DASH) score [24], and Simple Shoulder Test (SST) [25]. After excluding two studies without sufficient data for the analysis [9, 20], data syntheses were conducted based on six studies [10, 16–19, 21] and showed statistically significant differences in functional recovery in favour of the application of FSA (CMS: MD 5.07; 95% CI 3.40–6.74; *I*^*2*^ = 0.00%; 95% PI 2.361–7.78; ASES: MD 5.08; 95% CI 3.67–6.49; *I*^*2*^ = 0.00%; 95% PI 1.98–8.18), without obvious evidence of heterogeneity (Fig. 3).

### Radiographic assessments

**Fig. 4:**
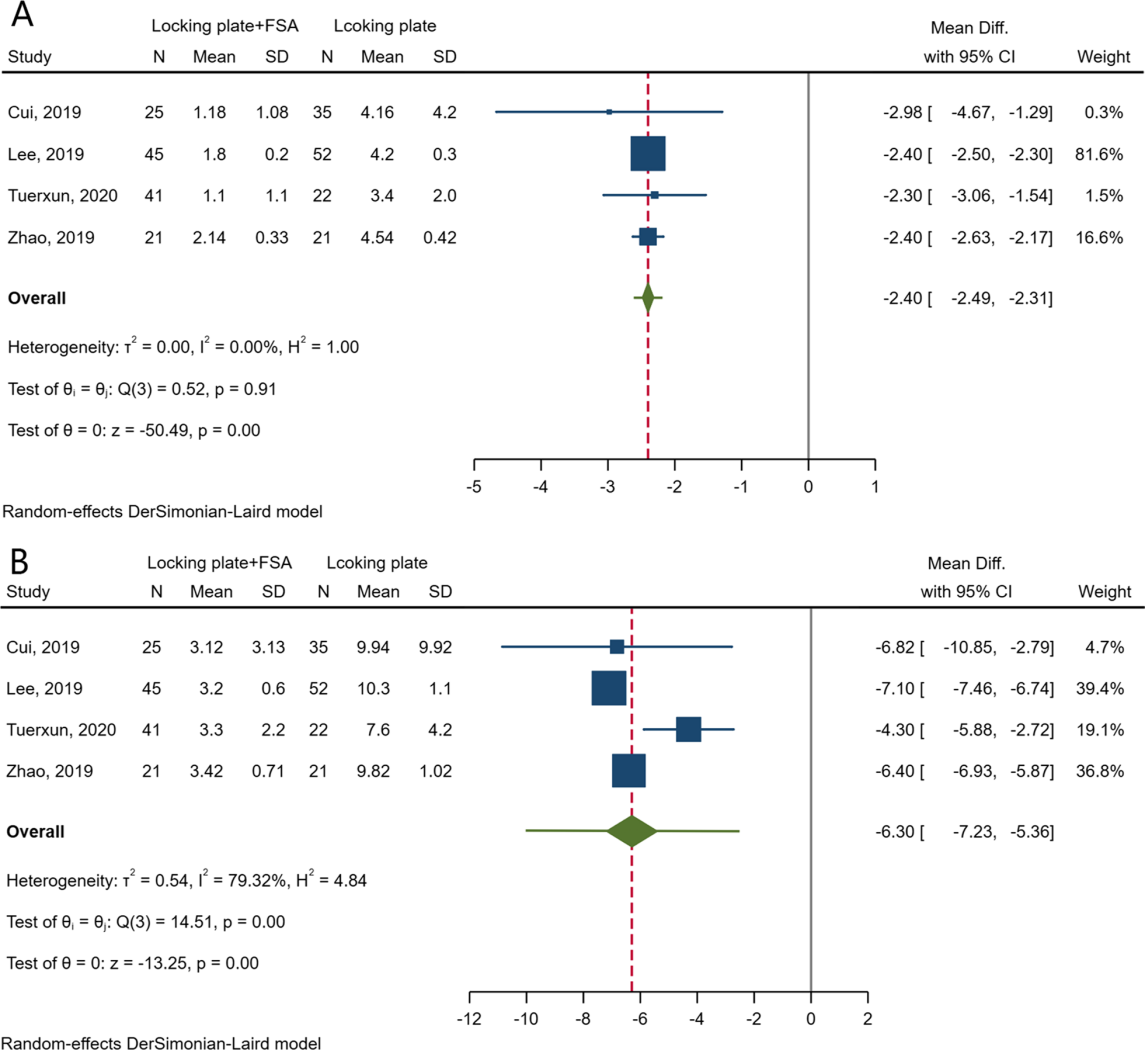
Forest plots of radiographical changes in postoperative HHH (**a**) and NSA (**b**)

Postoperative radiographic assessments were conducted in seven studies [9, 16–21]. The changes in humeral head height (HHH) and postoperative neck–shaft angle (NSA) were the most commonly used parameters in these trials and were reported in four articles, respectively. The data synthesis showed that the application of FSA was associated with fewer changes both in the HHH (MD -2.40; 95% CI − 2.49 to − 2.31; *I*^*2*^ = 0.00%; 95% PI − 2.61 to − 2.20) and in the NSA (MD − 6.30; 95% CI − 7.23 to − 5.36; *I*^*2*^ = 79.32%; 95% PI − 10.06 to − 2.53) (Fig. 4), indicating the beneficial effects of FSA in the maintenance of fracture reduction. High heterogeneity was detected in the analysis of the changes in NSA (*I*^*2*^ = 79.32%). Subsequent influence analyses were performed by removing an individual study by turns and did not find dramatic alterations both in either the heterogeneity or the pooled result, which confirmed the stability of the result. In addition, the 95% PI was entirely below zero, which was consistent with the meta-analytic result and was strong evidence indicating that the application of FSAs would be beneficial in reduction maintenance when applied in at least 95% of the individual study settings.

### Postoperative shoulder ROM

**Fig. 5:**
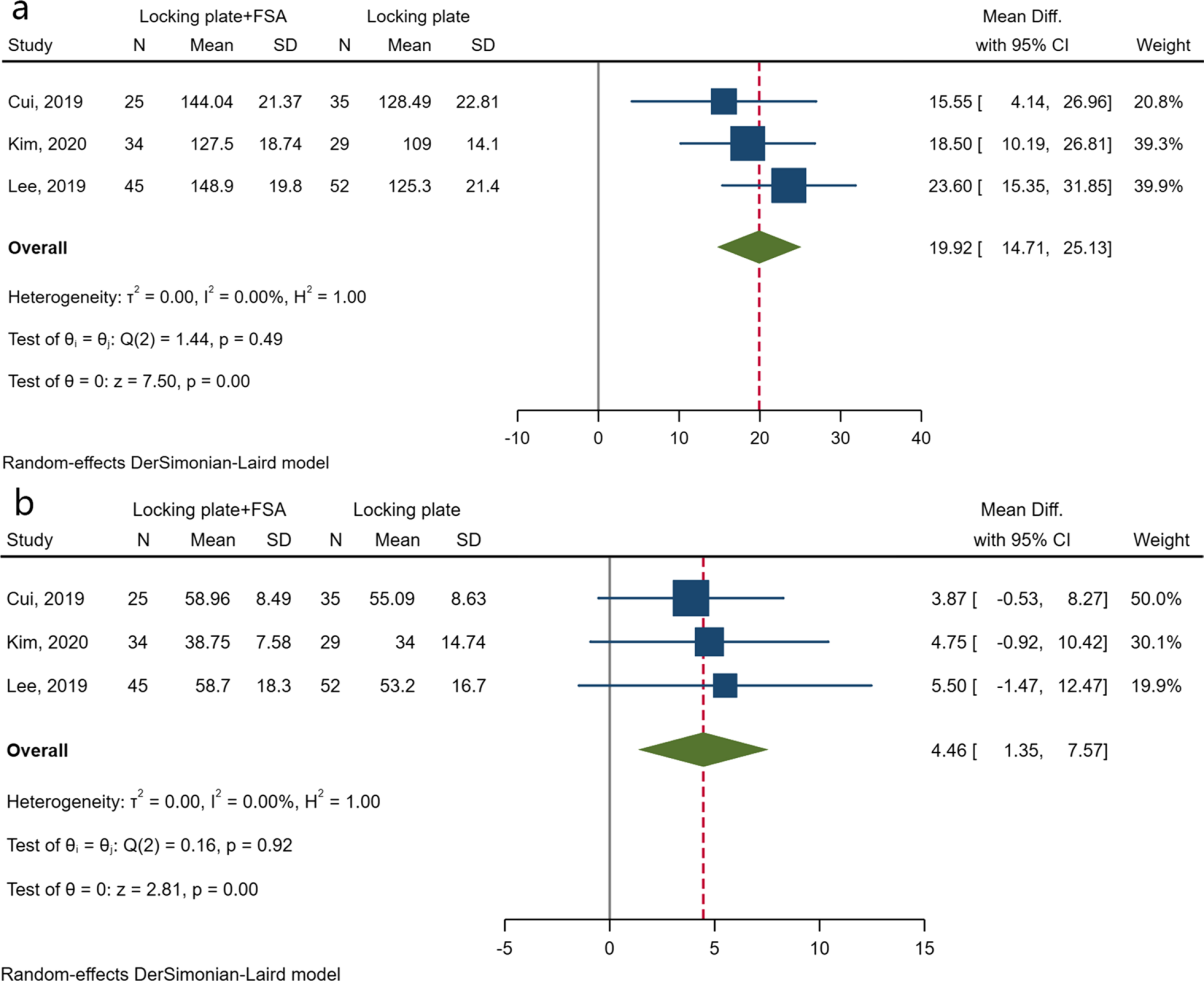
Forest plots of postoperative forward elevation (**a**) and external rotation (**b**)

Postoperative ROM was reported in four articles [10, 16–18], and the detailed results are presented in Additional file 3. One article was excluded from data synthesis, as the results in this article were reported in the form of “mean (range)” and no response was received from the authors after repeated inquiries [10]. Pooled analyses estimated statistical significance in terms of forward elevation (MD 19.92; 95% CI 14.71 to 25.13; *I*^*2*^ = 0.00%; 95% PI − 13.85–53.68), and external rotation (MD 4.46; 95% CI 1.35 to 7.57; *I*^*2*^ = 0.00%; 95% PI − 15.70–24.62) (Fig. 5).

Only two articles were eligible for the meta-analysis about abduction, and a significant difference favouring the FSA group was reported in both of them [16, 17]. However, the mean value of the abduction angle differed enormously in these two trials (122.37° ± 22.31° vs. 140.64° ± 20.34° in Cui 2019; 48.25° ± 17.71° vs. 118.25° ± 17.49° in Kim 2020), and high heterogeneity was observed in the data synthesis (MD 44.25; 95% CI -6.44 to 94.94; *I*^*2*^ = 98.07%). Given the high between-study heterogeneity and small number (*n* = 2) of included studies in this analysis, this heterogeneous body of studies might be too limited to make firm conclusions about postoperative abduction.

### Intraoperative information

Intraoperative blood loss and surgical duration were reported in one article, respectively [9, 17]. Due to the limited number of articles, data synthesis was not undertaken, and the results are summarized in Table 2. No difference was noted in terms of intraoperative blood loss according to Chen [9]. An increased likelihood of operative duration associated with the application of FSAs was detected in Kim [15].

**Table 2:** Outcomes of postoperative ROM and interoperative information

Outcome	Study	Findings		*P* value
		Locking plate + FSA	Locking plate	
Intraoperative blood loss (ml)	Chen [9]	232.77	219.52	0.332
Surgical duration (min)	Kim [15]	92.65 ± 19.25	76.65 ± 17.32	0.016

### Small-study effects

Due to the limited number of studies included in each comparison, the small-study effect was not routinely assessed in all the comparisons. The result of Egger’s test suggested that there were no obvious small-study effects in the comparison of overall complications, the rate of patients with orthopaedic complications, and the functional scores of CMS. These results are given in Additional file 4. Similarly, due to the limited number of included studies, the conclusions from Egger’s test were not robust enough.

## Discussion

This meta-analysis including eight trials showed that, compared with the use of locking plates alone, the combination of locking plates and FSAs could yield a reduction in postoperative complication incidence and improved outcomes regrading radiographic findings, functional recovery, postoperative forward elevation, and external rotation. However, no firm conclusions could be drawn regarding the postoperative abduction and internal rotation.

The theoretical advantage of using a fibular strut is to act as both an indirect reduction tool and a mechanical support for proximal humeral fractures with varus malalignment and a disrupted medial column, thus facilitating fracture reduction and decreasing the risk of postoperative complications [26]. Sometimes it even serves as a salvage protocol for fractures with severe metaphyseal comminution and osteoporosis. As shown in Fig. 2, data synthesis indicated that the application of FSA leads to the likelihood of reducing the risk of complications, with little heterogeneity. This result should be interpreted with caution since there was a lack of uniformity in monitoring and reporting these adverse events between trials. The subsequent analysis regarding the rate of patients with orthopaedic complications could be deemed as a sensitive analysis in some way, which tried to eliminate the heterogeneity between studies. Another concern is the additional risk of allograft-related complications such as rejection. However, they were not observed in the included studies.

Pooling the estimates of postoperative radiographic parameters suggested the beneficial effect of FSAs in the maintenance of fracture reduction. Although considerable heterogeneity was detected in the analysis of radiographical change in NSA, no special finding was noted in the influence analysis of individual studies, and the 95% prediction interval was consistent with the result of data synthesis. The prediction interval can be calculated when the meta-analysis contains at least 3 articles and is a predictor indicating the treatment effect in an individual setting. It can make the analysis more useful in clinical practice and decision-making. In this meta-analysis, the 95% prediction interval was entirely below zero, which indicates that FSA will be beneficial when applied in at least 95% of the individual study settings.

By reviewing current available evidence [8, 9, 16, 20, 21], we noticed that there were many ways to implant the fibular strut: (I) placing it adjacent to the medial cortex (Gardner’s method); (II) inserting it into the middle of the medullary cavity; and (III) paralleling to the calcar screw to support the medial hinge. Wang et al. retrospectively analysed the data from 128 patients with 4-part proximal humeral fractures, and they found that the clinical outcomes of the observation group, in which the fibular segment was implanted parallel to the calcar screw, were comparable to those of a matched group using Gardner’s method [20]. But till now, no study has directly compared and analysed whether there were differences between the abovementioned patterns I and II, and how these differences would affect the mechanical stability.

It is believed that the geometrical shape and mechanical strength of a fibular strut are appropriate for proximal humeral fractures. Inserting the fibular strut adjacent to the medial cortex of the humerus could provide immediate structural continuity and stability at the fracture site [8, 27]. Hypothetically, when the fibular segment is just intramedullary grafted, it can provide vertical sustentation to the humeral head. As shown in the figure of Additional file 5, the fibular strut and the locking screws that penetrated it could be regarded as a variation of an intramedullary nail that could supply some assisting force for the medial column. The risk of screw breakage is also decreased as the force arms of locking screws are shortened, which was initially the distance between the screw heads and plate but is now the distance between the screw heads and the cortical purchase of the fibula. Of course, sufficient clinical or biomechanical studies are needed to verify this presumption.

This is not the first systematic review about the utilization of FSAs in the management of proximal humerus fractures. However, to our knowledge, this is the first comprehensive meta-analysis of this issue. Two systematic reviews that did not include meta-analysis have been published. Biermann et al. used the relevant biomechanical and clinical studies to conduct a qualitative analysis about the augmentation of plate osteosynthesis for proximal humeral fractures [28]. The review by Saltzman et al. included four trials that had no control group [26]. Comparative analyses on the advantages and limitations of FSAs are still missing. The present meta-analysis, therefore, could be deemed as a supplement and an update to existing evidence.

Although the findings of this review support to the utilization of fibula allografts in the surgical management of proximal humeral fractures, there are still several limitations that should be of concern. This review was conducted based on non-RCTs with small sample sizes. It inevitably has a risk of selection bias. Most included trials confirmed the presence of an unstable medial column, but they drew their conclusions based on mixed fracture types (2-, 3- or 4-part fracture) and patterns (varus or valgus). This might be a potential source of the between-study heterogeneity and made it impossible to conduct subgroup analyses based on these items. Otherwise, this meta-analysis would be much more useful if similar fracture types or patterns (varus to varus and valgus to valgus) were compared. Other concerns are the drawbacks of FAS, which include the high cost and complexity of procedures [6, 10, 20]. Furthermore, the fibular strut might be an additional obstacle to the implantation of the shoulder prosthesis, which is the solution for the failure of fixation and HHAVN. All of these factors might restrict its wide application.

## Conclusion

For patients with an unstable medial column, findings based on the current evidence lend support to a favourable association of the FSAs in reducing postoperative complications, enhancing the maintenance of fracture reduction, improving functional recovery, and improving the postoperative ROM in terms of forward elevation and external rotation.

## Supplementary Information

**Additional file 1.** Search strategy for Pubmed.

**Additional file 2.** Results of quality assessments of included studies.

**Additional file 3.**  Results of postoperative ROM.

**Additional file 4.** Results of the Egger’s test.

**Additional file 5.** An illustration of the hypothesis about the mechanical mechanism of the intramedullary-grafted fibular segment.

## Data Availability

The datasets used and/or analysed during the current study are available from the corresponding author on reasonable request.

## Abbreviations

ASES: American Shoulder and Elbow Surgeons
CMS: Constant–Murley score
CR: Confident interval
DASH: Disability of arm–shoulder–hand
FSA: Fibular strut augmentation
HHAVN: Humeral head avascular necrosis
HHH: Humeral head height
MD: Mean difference
MeSH: Medical Subject Headings
NOS: Newcastle–Ottawa scale
NSA: Neck–shaft angle
OR: Odds ratio
PI: Prediction interval
PRISMA: Preferred Reporting Items for Systematic Reviews and Meta-Analyses
RCT: Randomized controlled trials
ROM: Range of motion
SST: Simple Shoulder Test
