# Supplementary material for: Effects of fibular strut augmentation for the open reduction and internal fixation of proximal humeral fractures: a systematic review and meta-analysis
Source: J Orthop Surg Res. 2022 Jun 21;17:322. doi: 10.1186/s13018-022-03211-4 (PMC9210738; doi:10.1186/s13018-022-03211-4)
Supplement: Supplementary file 1 — Additional file 1. Search strategy for Pubmed. [file 13018_2022_3211_MOESM1_ESM.pdf]

Additional file 1. Search strategy for Pubmed.

#1 “Humerus” [Mesh]
#2 Humeral [tiab]
#3 #1 OR #2
#4 “Fibula” [Mesh]
#5 (Fibular [tiab]) OR (Fibulas [tiab])
#6 #4 OR #5
#7 Eng [Language]
#8 Filters: from 2008 to 2021
#9 #3 AND #6 AND #7 AND #8
