# Supplementary material for: Effects of fibular strut augmentation for the open reduction and internal fixation of proximal humeral fractures: a systematic review and meta-analysis
Source: J Orthop Surg Res. 2022 Jun 21;17:322. doi: 10.1186/s13018-022-03211-4 (PMC9210738; doi:10.1186/s13018-022-03211-4)
Supplement: Supplementary file 2 — Additional file 2. Results of quality assessments of included studies. [file 13018_2022_3211_MOESM2_ESM.pdf]

Additional file 2. Results of quality assessments of included studies.

Study ID	Newcastle-Ottawa Scale		
	Selection	Comparabilty	Outcome
Chen 2018	★★★★	★☆	★☆☆
Cui 2019	★★★☆	★☆	★☆☆
Davids 2020	★★★☆	★★	★☆★
Kim 2020	★★★★	★☆	★☆★
Lee 2019	★★★★	★★	★☆★
Wang 2019	★★★☆	★★	★☆★
Zhao 2019	★★★☆	★★	★☆★
Tuerxun 2020	★★★★	★★	★☆★
