# Supplementary material for: Effects of fibular strut augmentation for the open reduction and internal fixation of proximal humeral fractures: a systematic review and meta-analysis
Source: J Orthop Surg Res. 2022 Jun 21;17:322. doi: 10.1186/s13018-022-03211-4 (PMC9210738; doi:10.1186/s13018-022-03211-4)
Supplement: Supplementary file 3 — Additional file 3. Results of postoperative ROM. [file 13018_2022_3211_MOESM3_ESM.pdf]

Additional file 3. Results of postoperative ROM.

Outcome	Study ID	Findings		P value
		FSA+LCP	LCP	
Forward elevation (degree)	Cui 2019	144.04±21.37	128.49 ± 22.81	0.010
	Davids 2020	142.3（110-160）	140.3（70-180）	1.000
	Kim 2020	127.50 ± 18.74	109.00 ± 14.10	0.001
	Lee 2019	148.9 ± 19.8	125.3 ± 21.4	0.042
Abudtion (degree)	Cui 2019	140.64 ± 20.34	122.37 ± 22.31	0.002
	Davids 2020	125(90-160)	120.6(70-170）	0.800
	Kim 2020	118.25 ± 17.49	48.25 ± 17.71	0.000
External rotation (degree)	Cui 2019	58.96 ± 8.49°	55.09 ± 8.63°	0.090
	Davids 2020	54.2（20-80）	51.5（5-80）	0.800
	Kim 2020	38.75 ± 7.58	34.00 ± 14.74	0.341
	Lee 2019	58.7 ± 18.3	53.2 ± 16.7	0.175
Internal rotation (degree)	Cui 2019	T12 level (L5-T5)	L1(buttock-T5)	0.438
	Kim 2020	L5	L1	0.000
