# Supplementary material for: Effects of fibular strut augmentation for the open reduction and internal fixation of proximal humeral fractures: a systematic review and meta-analysis
Source: J Orthop Surg Res. 2022 Jun 21;17:322. doi: 10.1186/s13018-022-03211-4 (PMC9210738; doi:10.1186/s13018-022-03211-4)
Supplement: Supplementary file 4 — Additional file 4. Results of the Egger’s test. [file 13018_2022_3211_MOESM4_ESM.pdf]

Additional file 4. Results of the Egger’s test

Parameter	Number of included studies	Egger's test		
		Intercept	95% CI	P >
Overall complications	8	0.273	-1.837 to 2.383	0.762
Rate of patients with orthopedic complications	6	1.447	-1.205 to 4.100	0.204
CMS	5	0.498	-1.013 to 2.009	0.412
