# Supplementary material for: Effects of fibular strut augmentation for the open reduction and internal fixation of proximal humeral fractures: a systematic review and meta-analysis
Source: J Orthop Surg Res. 2022 Jun 21;17:322. doi: 10.1186/s13018-022-03211-4 (PMC9210738; doi:10.1186/s13018-022-03211-4)
Supplement: Supplementary file 5 — Additional file 5. An illustration of the hypothesis about the mechanical mechanism of the intramedullary-grafted fibular segment. [file 13018_2022_3211_MOESM5_ESM.pdf]

Additional file 5.

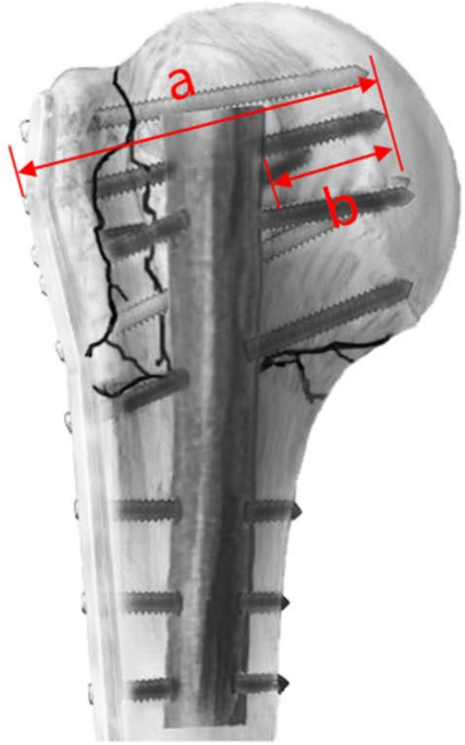

The fibular cortical segment and the traversed locking screws could be regard as a variation of an intramedullary nail. Along the lines of these screws, this “intramedullary nail” could provide support force to the medial column by the lever arm effect. Besides, the distances of the force arms of locking screws were also shortened (from a to b), which might reduce the risk of screw breakage.
